# Regulation of Cardiac Contractility by the Alpha 2 Subunit of the Na^+^/K^+^-ATPase

**DOI:** 10.3389/fphys.2022.827334

**Published:** 2022-06-22

**Authors:** Jonas Skogestad, Jan Magnus Aronsen

**Affiliations:** ^1^ Department of Molecular Medicine, Institute of Basic Medical Sciences, Faculty of Medicine, University of Oslo, Oslo, Norway; ^2^ Department of Pharmacology, Oslo University Hospital, Oslo, Norway

**Keywords:** NKA, sodium, calcium, ion gradients, NCX, Cardiomyocyte, EC-coupling

## Abstract

Cytosolic Na + concentrations regulate cardiac excitation-contraction coupling and contractility. Inhibition of the Na^+^/K^+^-ATPase (NKA) activity increases cardiac contractility by increasing cytosolic Ca^2+^ levels, as increased cytosolic Na^+^ levels are coupled to less Ca^2+^ extrusion and/or increased Ca^2+^ influx from the Na^+^/Ca^2+^-exchanger. NKA consists of one α subunit and one β subunit, with α1 and α2 being the main α isoforms in cardiomyocytes. Substantial evidence suggests that NKAα2 is the primary regulator of cardiac contractility despite being outnumbered by NKAα1 in cardiomyocytes. This review will mainly focus on differential regulation and subcellular localization of the NKAα1 and NKAα2 isoforms, and their relation to the proposed concept of subcellular gradients of Na^+^ in cardiomyocytes. We will also discuss the potential roles of NKAα2 in mediating cardiac hypertrophy and ventricular arrhythmias.

## Introduction

Cardiac contraction is initiated by the opening of voltage-gated Na^+^ channels, leading to rapid Na^+^ influx into cardiomyocytes and membrane depolarization. The membrane depolarization activates the L-type Ca^2+^ channels, and the resulting Ca^2+^ influx leads to the opening of the ryanodine receptors (RyRs), causing a substantial Ca^2+^ release from the sarcoplasmic reticulum (SR). The ensuing rise in cytosolic [Ca^2+^] eventually leads to cardiac contraction when free cytosolic Ca^2+^ binds troponin-C in the myofilaments, where the contraction’s strength depends on the levels of cytosolic Ca^2+^ (Bers, 2002). For the cardiomyocyte to relax and re-lengthen, the Ca^2+^ entering the cytosol during the excitation-contraction coupling must be extruded from the cytosolic space. The Ca^2+^ extrusion is mainly executed by either the SR Ca^2+^ ATPase (SERCA2), which pumps Ca^2+^ into the SR, or the Na^+^/Ca^2+^- exchanger (NCX), which extrudes Ca^2+^ over the cell membrane in exchange for 3 Na^+^ ions.

Because NCX exchanges Ca^2+^ for Na^+^, NCX indirectly couples intracellular [Na^+^] to regulate cardiac contractility. NCX can operate in two modes: A forward mode with Ca^2+^ efflux and Na^+^ influx and a reverse mode with Ca^2+^ influx and Na^+^ efflux. Whether NCX operates in forward or reverse mode depends on the transmembrane gradients for Na^+^ and Ca^2+^ and the membrane potential. Most of the time, NCX extrudes Ca^2+^ (forward mode), and Ca^2+^ entry through NCX (reverse mode) only occurs briefly during the early stages of a regular action potential, where increased [Na^+^]_i_ (due to the Na^+^ influx in early phases of the action potential), low [Ca^2+^]_i_, and positive membrane potential all favors NCX-mediated Ca^2+^ influx (Bers, 2001).

During a regular contraction-relaxation cycle, a considerable amount of Na^+^ enters the cell, mainly through the Na^+^ channels and the NCX (Bers and Despa, 2009). To maintain equilibrium, all Na^+^ entering the cell must be transported out of the cell. The main Na^+^ efflux route is the Na^+^/K^+^-ATPase (NKA), which uses the energy from ATP to extrude 3 Na^+^ for 2 K^+^. NKA is ubiquitously expressed in all cell types and contributes to a wide range of cellular tasks in addition to the regulation of cardiac contractility, including secondary active transport, volume regulation, and pH regulation (Kaplan, 2002). NKA is a protein complex consisting of an α subunit and a β subunit that form a “minimal functional unit” and a Ƴ subunit (FXYD proteins) that regulates NKA function. Different subunit isoforms (α1-3 and β1-2) can form different αβ combinations in the heart. In humans, NKAα1-3 is expressed in all heart tissue (Sweadner et al., 1994), while β1 is the predominant β isoform (Liu et al., 2016). The α1β1-combination of NKA is the most abundant and has been extensively studied (McDonough et al., 1990; Blanco and Mercer, 1998). NKAα1 and NKAα2 are expressed in the left ventricle of adult rodents (Orlowski and Lingrel, 1988; Hensley et al., 1992; James et al., 1999), possibly with some expression of NKAα3 (Harada et al., 2006; Stanley et al., 2015). Protein levels, mRNA expression, and functional experiments suggest that NKAα1 is the most abundant cardiac isoform (70–95%) in humans, large animals, and rodents, with consistently lower expression and activity of NKAα2 (10–30%) (Sweadner et al., 1994; Gao et al., 1999; Berry et al., 2007; Swift et al., 2007). Despite being outnumbered by a factor of approximately 10:1, the existing evidence clearly suggests that NKAα2 and not NKAα1 is the primary regulator of cardiac contractility. We here aim to review the evidence of NKAα2 as regulator of cardiac contractility, and discuss possible underlying mechanisms and pathophysiological roles of NKAα2-mediated control of cardiac excitation-contraction coupling.

### NKA/NCX Interaction as a Regulator of Intracellular [Ca^2+^] and Cardiac Contractility

NKA indirectly regulates NCX activity through a functional interaction: NKA regulates cytosolic [Na^+^], thereby modulating NCX activity and subsequently cytosolic [Ca^2+^] and cardiac contractility. A key, unsolved question is whether NCX activity is regulated by the average [Na^+^] and [Ca^2+^] in the cytosol or whether NCX “senses” localized Na^+^ pools, i.e., subdomains where the [Na^+^] is higher or lower than the average [Na^+^]. Such localized gradients are well documented for Ca^2+^ ions, particularly in the dyad, where repetitive Ca^2+^ influx causes standing and dynamic gradients of Ca^2+^ between the dyad and the bulk cytosol (Bers, 2008; Acsai et al., 2011).

The existence of localized Na^+^ gradients in cardiomyocytes is more controversial and the evidence conflicting (Garcia et al., 2016; Lu and Hilgemann, 2017). The lack of methods allowing high-resolution measurements of intracellular Na^+^ means that the proposed concept of localized Na^+^ gradients is based mainly on indirect evidence. Perhaps most profound among these indirect lines of evidence is the large amount of data showing that NKAα2 preferentially regulates cardiac contractility without modulating global levels of Na^+^.

#### NKAα2 Preferentially Regulates Ca^2+^ Cycling and Cardiac Contractility

Different cardiac NKAα isoforms are present in nearly all species, including humans and rodents (Hensley et al., 1992; Sweadner et al., 1994), suggesting differential functional roles in the heart, which is also supported by the fact that the isoform-defining regions are highly conserved through evolution (Baxter-Lowe et al., 1989; Pressley, 1992). Mice lacking both copies of the NKAα1 gene die during the embryonical stage, while mice without NKAα2 die immediately following birth (James et al., 1999; Barcroft et al., 2004; Dostanic-Larson et al., 2006). In a landmark paper, James *et al.* showed that heterozygous inactivation of NKAα2 (NKAα2^+/-^) increased Ca^2+^ transients and cardiac contractility in mice, while NKAα1^+/-^ mice were hypocontractile (James et al., 1999). Genetic analysis of the NKAα1^+/-^ mice revealed alterations in several genes important for ion transport and cardiac contractility (Moseley et al., 2005), and the functional effects in NKAα1 deficient mice thus might be due to indirect effects. However, these initial findings suggesting a distinct role of NKAα2 in regulating NCX activity, intracellular [Ca^2+^], and cardiac contractility has since been reproduced and elaborated by several groups (Yamamoto et al., 2005; Swift et al., 2007; Swift et al., 2010; Despa et al., 2012).

The glycoside ouabain has been an invaluable tool to evaluate functional roles of NKA α1 versus NKAα2. NKAα1 in rodents is less sensitive to the glycoside ouabain than NKAα2 due to two positively charged amino acids (arginine-111 and aspartic acid-122) in the extracellular region (Price and Lingrel, 1988; Price et al., 1990) of NKAα1. Ouabain does not alter the NKAα1 activity itself (Dostanic-Larson et al., 2006) or the coefficients towards Na^+^ and K^+^ (Periyasamy et al., 1983). In contrast, NKAα2 has a higher affinity towards ouabain (O'Brien et al., 1994; Ishizuka et al., 1996). A double-sigmoid affinity curve is seen in mice and rats (Swift et al., 2007; Despa et al., 2012), allowing specific inhibition of NKAα2 with a low dose of ouabain (300 nM), where only a small fraction of NKAα1 is inhibited. Specific inhibition of NKAα2 increases NCX-sensed [Na^+^], increases Ca^2+^ transient amplitude and cardiac contractility without effects on global [Na^+^] (Yamamoto et al., 2005; Swift et al., 2007; Despa et al., 2012).

Overexpression of NKAα1 and NKAα2 both lower intracellular [Ca^2+^], but overexpression of NKAα1 reduces the expression of NKAα2 and *vice versa* (Correll et al., 2014). Generating SWAP mice has helped overcome these limitations of NKAα1 and NKAα2 overexpression. The SWAP mice have reversed NKAα isoform affinity towards ouabain, i.e., NKAα1 is ouabain-sensitive, and NKAα2 is ouabain-resistant, while the expression of both NKAα isoforms remains unaltered (Dostanic et al., 2003). This model has generated some apparently divergent findings. In contrast to many previous reports, Dostanic *et al.* found that NKAα1 interacted with NCX and regulated cardiac contractility when ∼40% of NKAα1 was inhibited (Dostanic et al., 2004). On the other hand, a later study found that 25% NKA inhibition in the SWAP mice (i.e., NKAα1 inhibition) and 25% NKA inhibition in the WT mice (i.e., NKAα2 inhibition) gave a similar rise in intracellular [Na^+^], but only WT mice with NKAα2 inhibition exerted increased Ca^2+^ levels (Despa et al., 2012). While these results could seem contradictory, one should bear in mind that the question should be whether NKAα2 *preferentially* regulates Ca^2+^ cycling and contractility compared to NKAα1, and not whether NKAα1 inhibition is without any effect.

The affinity of the clinically used glycoside digoxin is up to four-fold higher towards human NKAα2 compared to NKAα1 (Katz et al., 2010), and glycoside-induced inotropy and hypertension have been shown to be mediated by NKAα2 in mice (Dostanic et al., 2003; Dostanic et al., 2005). Moreover, NKAα2 preferentially regulates Ca^2+^ cycling in both astrocytes (Golovina et al., 2003) and smooth muscles (Zhang et al., 2005). Overall, existing evidence clearly suggest that the more abundant NKAα1 has a “housekeeping” role, regulating global [Na^+^] (Aronsen et al., 2013), whereas NKAα2 could specifically regulate Na^+^ in distinct functional domains shared with NCX in cardiomyocytes.

### Mechanisms for Regulation of Cardiac Contractility by NKAα2

**FIGURE 1:**
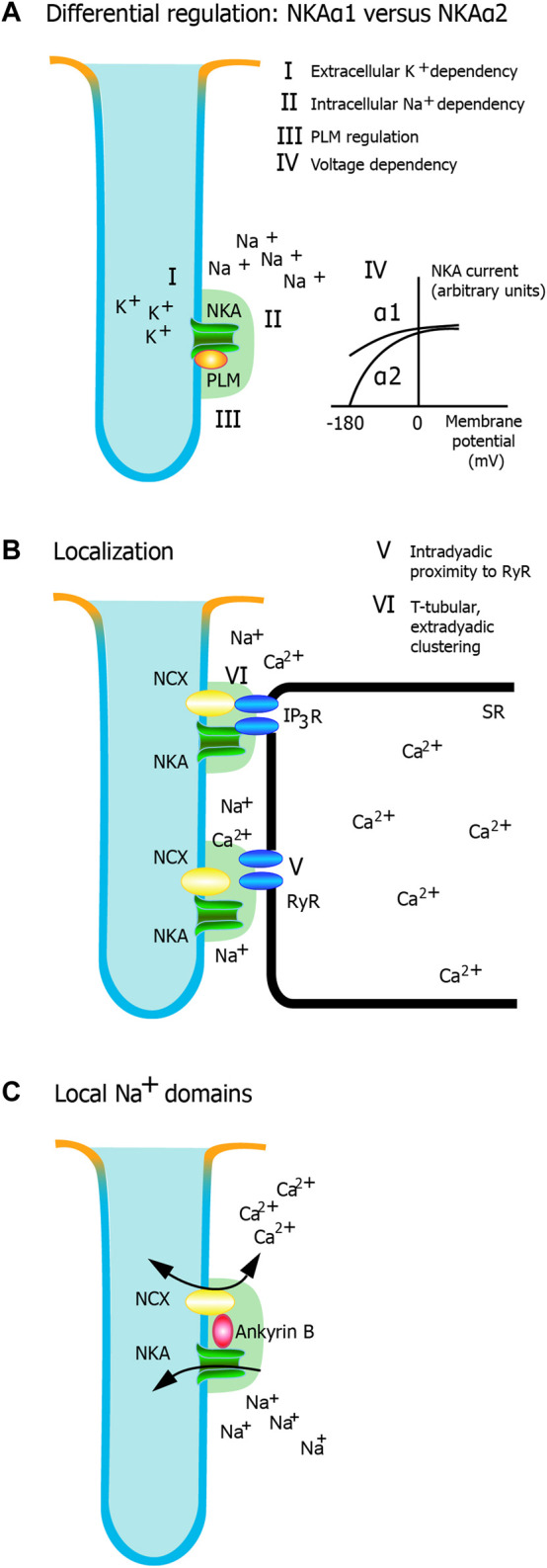
Potential mechanisms for regulation of cardiac contractility by NKAα2. Different mechanisms have been proposed, and this figure highlights the most important hypotheses. **(A)** Differential regulation of α isoforms. NKA activity is regulated by **(I)** the extracellular K^+^ dependency of NKA, ii) the intracellular Na^+^ dependency of NKA, iii) beta-adrenergic regulation mediated through phospholemman, and iv) voltage dependency. Only the extracellular K^+^ dependency and the voltage dependency are markedly different between NKAα1 and NKA α2. **(B)** Localization. There is a relative clustering of NKAα2 in the t-tubules. v) Intradyadic proximity to RyR and vi) T-tubular, extradyadic clustering (potentially interacting with IP3 receptors) are two mechanisms that could have an impact on dyadic Ca^2+^ signaling. Whether one or both are important for NKAα2 regulation of Ca^2+^ and cardiac contractility remain to be established. c) Local Na^+^ domains. AnkyrinB facilitates a macromolecular complex with NKA and NCX, characterized by tight regulation of Na^+^ and Ca^2+^ in local domains.

While there is a paucity of conclusive evidence, several structural and molecular mechanisms have been proposed as to how NKAα2 preferentially regulates NCX activity and cytosolic Ca^2+^ fluxes in cardiomyocytes. An illustration of the main hypotheses are shown in Figure 1, and the different suggested mechanisms will be discussed in the next sections.

#### Differential Regulation of NKA α Isoforms

The primary regulators of NKA activity are extracellular [K^+^], intracellular [Na^+^], the inhibitory protein phospholemman, and the membrane potential, and we will here discuss each of these separately with focus on any differences NKAα2 versus NKAα1.

##### Intracellular [Na^+^] and Extracellular [K^+^] Affinity

There is a sigmoid relationship between intracellular [Na^+^] and the NKA current in cardiomyocytes, where increasing concentrations of Na^+^ lead to increased NKA currents (Nakao and Gadsby, 1989; Skogestad et al., 2019a). No differences in the Na^+^ dependence between NKAα1 and NKAα2 have been found (Price et al., 1990; Berry et al., 2007; Bibert et al., 2008). In contrast, the extracellular [K^+^] dependency differs between NKAα1 and NKAα2 (Crambert et al., 2002; Bibert et al., 2008; Han et al., 2009). NKAα1 has higher affinity towards extracellular [K^+^] (k_0.5_=1.5 mM) compared to NKAα2 (k_0.5_=2.9 mM) (Han et al., 2009), meaning that NKAα1 is nearly maximally activated at physiological [K^+^] (∼4.5 mM). This could have some critical physiological and pathophysiological implications. Diffusion of K^+^ could be restricted in the T-tubules (Swift et al., 2006), which could influence the regulation of NKAα2 activity more than NKAα1, especially considering the relative abundance of NKAα2 in the T-tubules (Berry et al., 2007; Despa and Bers, 2007), as discussed in the next section. The different extracellular [K^+^] affinity could also be significant in hypokalemia, a common clinical condition that increases the risk of Ca^2+^-induced triggered arrhythmias (Kjeldsen, 2010; Weiss et al., 2017). As discussed later, we have previously shown that hypokalemia-induced effects on Ca^2+^ cycling and arrhythmias are mainly mediated through NKAα2 (Aronsen et al., 2015).

##### Phospholemman

Phospholemman (PLM) is a member of the FXYD family of small, membrane-spanning proteins (Palmer et al., 1991) that associates with the α subunit of the NKA (Khafaga et al., 2012). PLM binding to NKA increases the apparent affinity for intracellular [Na^+^] and extracellular [K^+^] (Crambert et al., 2002; Despa et al., 2005; Han et al., 2009), which reduces NKA activity. PLM mediates the effects of β-adrenergic signaling on NKA, and it has two main phosphorylation sites: Serine (Ser)-63 and Ser-68 (Walaas et al., 1994; Song et al., 2005). A third phosphorylation site at Threonine (Thr)-69 has also been described (Fuller et al., 2009), but phosphorylation of Ser-63 and Ser-68 is sufficient to mediate all functional effects (Han et al., 2010) of beta-adrenergic activation. Ser-68 is the primary substrate for protein kinase A (PKA) (Silverman et al., 2005), while protein kinase C (PKC) phosphorylates both Ser-63 and Ser-68 (Han et al., 2006; Fuller et al., 2009). Following PLM phosphorylation, PKA phosphorylation at Ser-68 increases NKA activity by reducing the affinity of intracellular [Na^+^] (Despa et al., 2005; Han et al., 2006; Bibert et al., 2008; Despa et al., 2008). Although PKC phosphorylation also consistently increases NKA activity, the evidence is equivocal on whether this is due to a reduction in the intracellular [Na^+^] affinity, an increase in the maximal activity, or both (Han et al., 2006; Bibert et al., 2008; Han et al., 2010).

Two early reports found no effects of β-adrenergic stimulation on NKAα2 activity (Silverman et al., 2005; Berry et al., 2007). In these studies, the NKAα2 activity was calculated by subtracting ouabain-sensitive current from the total current, a strategy that could lead to large variation and in our opinion, a high risk of false-negative results. Later studies found that PLM interacted with both NKAα1 and NKAα2 (Feschenko et al., 2003; Bossuyt et al., 2005; Bossuyt et al., 2009), that PKA-mediated PLM phosphorylation increased the activities of both NKAα1 and NKAα2 (Bibert et al., 2008; Bossuyt et al., 2009). The same studies also found that PKC regulated the maximal activity of NKAα2, not NKAα1, whereas the dependency of intracellular [Na^+^] was affected similarly in both isoforms (Bibert et al., 2008; Bossuyt et al., 2009).

##### Voltage-Dependence of NKAα2 and NKAα1

As NKA moves one positive charge out of the cell per pumping cycle (3 Na^+^ ions out and 2 K^+^ ions in), NKA activity becomes dependent on the membrane potential. The current-voltage relationship for NKAα2 is different compared to NKAα1. NKAα1 is activated over a broad range of physiological potentials, whereas NKAα2 is nearly inactive at resting membrane potentials, indicating that NKAα2 only is active during phases one to four of the action potential when the membrane potential is more positive (Swift et al., 2007; Stanley et al., 2015). Some suggest that NKAα2 might be acting as a “pump reserve”, where increased Na^+^ influx during the action potential are counteracted by more Na^+^ extrusion during the same period (Stanley et al., 2015). Another possible (and not mutually exclusive) interpretation is that Na^+^ influx during the contraction more readily accumulates in the NKAα2 compartment, rendering the baseline Na^+^ levels higher in the NKAα2 compartment than in the NKAα1 compartment. Thus, the differences in the voltage dependence of NKAα1 and NKAα2 could contribute to a unique local ion environment. However, several studies have shown preferential NKAα2 regulation of NCX also at fixed membrane potential (usually in the range from -50 mv to 0 mV) (Yamamoto et al., 2005; Swift et al., 2007; Skogestad et al., 2019b), indicating that differences in voltage-dependence alone are not sufficient to explain the preferential regulatory role of NKAα2.

#### NKA Localization

##### Subcellular Localization

A prerequisite for an effective excitation-contraction coupling is the dyads, i.e., functional Ca^2+^domains in the T-tubules where sarcolemmal L-type Ca^2+^ channels are located near ryanodine receptors (RyRs), allowing effective Ca^2+^-induced Ca^2+^ release into the cytosol (Bers, 2002; Louch et al., 2010). Thus, an attractive hypothesis would be that preferential localization of NKAα2 and NCX in cardiac T-tubules could allow local Ca^2+^ modulation in specific subcellular domains involved in the excitation-contraction coupling. Similar to NCX (Frank et al., 1992; Despa et al., 2003; Sipido et al., 2013), NKAα2 is indeed relatively more abundant in the T-tubules, as suggested by immunofluorescence (Mohler et al., 2005; Silverman et al., 2005), super-resolution microscopy (Yuen et al., 2017), and NKA current measurements in de-tubulated cardiomyocytes (Berry et al., 2007; Despa and Bers, 2007; Swift et al., 2007) [although one early study did not find this pattern (McDonough et al., 1996)]. NKAα2 is also highly clustered in the T-tubules in skeletal muscles, where it constitutes the main NKA isoform (Radzyukevich et al., 2013; DiFranco et al., 2015).

However, NKAα1 is also present in the T-tubules in cardiomyocytes (Mohler et al., 2005). Despite NKAα2 being *relatively* abundant in the T-tubules (i.e. high T-tubule/surface sarcolemma ratio), the total amount of NKAα1 in the T-tubules is equal to or even higher compared to NKAα2 despite a low T-tubule/surface sarcolemma ratio for NKAα1 (Berry et al., 2007; Despa and Bers, 2007; Swift et al., 2007). For instance, Swift *et al.* found that NKAα2 comprises 10% of the total NKA activity in rat cardiomyocytes, and 50% of the total NKAα2 activity was of T-tubular origin, indicating that about 5% of the total NKA activity was due to NKAα2 pumps located in the T-tubules. In contrast, only 10% of the total NKAα1 was located the T-tubules (Swift et al., 2007), but these data nevertheless indicate that NKAα1 outnumbers NKAα2 in the t-tubules with a 2:1 ratio.

NKAα2 could be more closely associated with RyR at SR junctional sites than NKAα1. Data from astrocytes, neurons, and smooth muscles suggest that NKAα2 assembles with NCX in a microdomain linked to the ER/SR (Juhaszova and Blaustein, 1997; Lencesova et al., 2004; Song et al., 2006). However, a recent study using super-resolution microscopy failed to show a similar arrangement in rat cardiomyocytes, as NKAα1 and NKAα2 were equally distant from RyRs (Yuen et al., 2017). As this study analyzed NKA clusters within 0.2–1 µm from RyR, while the distance between L-type Ca^2+^ channels and RyR in the dyad is 10–20 nm, later studies with higher resolution might reveal different RyR proximity for NKAα2 versus NKAα1.

##### Macromolecular Complexes

Ankyrins are a family of anchoring proteins that couples membrane proteins to the membrane cytoskeleton, and both NKA and NCX co-assemble with Ankyrin B. Ankyrin B apparently does not structurally discriminate between NKAα1 and NKAα2, as Ankyrin B is found to interact and co-localize with both NKAα1 and NKAα2 in the T-tubules, in addition to NCX and InsP3 receptors in a shared macromolecular complex (Mohler et al., 2003; Mohler et al., 2005). Interestingly, the Ankyrin B macromolecular complex with NKA and NCX seems to have an extradyadic localization, as neither Ankyrin B, NKAα1, NKAα2, nor NCX co-localize with RyR or L-type Ca^2+^ channels in cardiomyocytes (Mohler et al., 2005). One possibility is that the NKA/NCX domains, rather than being directly involved in the excitation-contraction coupling, indirectly regulate dyadic Ca^2+^ by modulating transsarcolemmal Ca^2+^ fluxes at the dyadic border, but more studies are needed to investigate this hypothesis.

#### Local Na^+^ Domains

Another possibility than differences in localization is that NKAα2 could, more tightly than NKAα1, regulate the local Na^+^ pools sensed by NCX. Any effect on Ca^2+^ cycling would be mediated through Na^+^, and differences in the ability to control local Na^+^ pools in the vicinity of NCX could potentially have significant effects on Ca^2+^ cycling and cardiac contractility *independently* of the localization of the shared NKA/NCX-domain.

Several studies suggest that NKAα2 preferentially regulates NCX-sensed Na^+^ and NCX activity. Yamamoto *et al.* first reported that local NCX-sensed (Na^+^) was higher in heterozygous NKAα2^+/-^ mice. Similarly, by using NKAα2 selective doses of oubain, Swift *et al.* later showed that NKAα2 regulated NCX-sensed (Na^+^) and NCX activity (Swift et al., 2007; Swift et al., 2010). Other molecular studies suggest that both NKAα1 and NKAα2 co-immunoprecipitate with NCX in cardiomyocytes (Dostanic et al., 2004; Mohler et al., 2005), which apparently represents a discrepancy to the idea of NKAα2 as a preferential regulator of NCX activity. However, no quantitative measurements on the degree of co-localization of NKAα1 versus NKAα2 with NCX have been performed, and data from other cell types indicate that NKAα2 more than NKAα1 interacts with NCX (Golovina et al., 2003; Lencesova et al., 2004). In addition, it is possible that the microarchitecture or functional features of the shared NKA/NCX macromolecular complexes are different between NKAα1 and NKAα2 in a way that is not assessed with the interaction assays. In support of this concept, even though it has been shown that Ankyrin B co-immunoprecipitates with both NKAα1 and NKAα2, we observed that disruption of NKA from Ankyrin B only affected local Na^+^ and NCX activity in the NKAα2 domains and not in the NKAα1 domains (Skogestad et al., 2019b).

#### Summary: Mechanisms for NKAα2 Mediated Regulation of Cardiac Contractility

In summary, there are several differences between NKAα1 and NKAα2 that could explain the observed role of NKAα2 as a regulator of cardiac contractility. NKAα2 is relatively abundant in the T-tubules of cardiomyocytes (Berry et al., 2007; Despa and Bers, 2007; Swift et al., 2007) and interacts with ER/SR junctions in other cell types (Juhaszova and Blaustein, 1997; Lencesova et al., 2004; Song et al., 2006), while it is questioned whether NKAα2 is more densely co-localized with the dyad in cardiomyocytes (Yuen et al., 2017). Regardless of localization, NKAα2 controls NCX-sensed Na^+^ levels and subsequently NCX activity, excitation-contraction coupling, and contractility (Yamamoto et al., 2005; Swift et al., 2007). In addition, both the voltage-dependence and the extracellular [K^+^] dependency are different between NKAα2 and NKAα1, possibly contributing to the ability of NKAα2 to regulate Ca^2+^ fluxes in cardiomyocytes.

### Role of Subsarcolemmal Na^+^ Gradients

Any effect of NKAα2 on intracellular [Ca^2+^] and cardiac contractility must be mediated through the regulation of intracellular [Na^+^], proposedly by altering local [Na^+^] in specific domains. In other words, a given change in NKA activity leads to alterations in local [Na^+^] sensed by NCX in the same compartment, with a more negligible (or no) effect on the [Na^+^] in more distant compartments. A prerequisite for this hypothesis is the presence of intracellular Na^+^ gradients between different compartments in cardiomyocytes.

The first reports of a subsarcolemmal space of Na^+^, i.e., a distinct submembrane compartment where Na^+^ is different from bulk cytosolic [Na^+^], came from a landmark paper by Leblanc and Hume (Leblanc and Hume, 1990). They observed that Na^+^ current activation was sufficient to induce Ca^2+^-induced Ca^2+^ release (CICR) through activation of reverse NCX, a mechanism that localized elevations of [Na^+^] in an undefined compartment coined “fuzzy space” (Lederer et al., 1990). Accumulation of Na^+^ in submembrane compartments has also been observed in compartments not directly involved in CICR, e.g., around NKA (Su et al., 1998; Despa and Bers, 2003; Silverman, 2003; Despa et al., 2004; Swift et al., 2007; Despa et al., 2012), and the broader term “subsarcolemmal space” is frequently used to encompass a submembrane compartment with differential [Na^+^] than the cytosol (Aronsen et al., 2013). We recently reported data that indicates that [Na^+^] is different between the Na^+^ channel compartments and the NKA compartments, arguing against a uniform distribution of Na^+^ throughout the subsarcolemmal space. These data suggest rather that Na^+^ is differentially regulated in distinct submembrane compartments (Skogestad et al., 2019a), i.e., “hotspots” and “coldspots” with higher or lower [Na^+^] than the cytosolic [Na^+^]. In support of a shared NKA/NCX compartment, another study observed that the subsarcolemmal [Na^+^] is similar for NKA and NCX after manipulation of the Na^+^ current (Su et al., 1998).

If such “hotspots” and “coldspots” exist, a fundamental question is how the proposed Na^+^ gradients could be generated and maintained. Na^+^ diffuses rapidly in the cytoplasm (Kushmerick and Podolsky, 1969), and similar diffusion kinetics in the subsarcolemmal space would lead to rapid dissipation of all Na^+^ gradients. Calculations show that to maintain the accumulation of Na^+^ in the subsarcolemmal space, the diffusion rates need to be 100-10,000 times slower than what is observed experimentally (Despa and Bers, 2003; Despa et al., 2004). It is possible that physical restrictions (e.g., membrane tortuosity, molecular and organelle crowding) and negative submembrane charges impede the free diffusion of ions.

A crucial aspect is the temporal duration of the proposed subsarcolemmal Na^+^ gradients. Weber *et al.* observed that Na^+^ current activation generates transient Na^+^ accumulation near NCX early during the action potential. Due to the positive membrane potentials and the general Na^+^ accumulation during the early phase of the action potential, NCX operates in reverse mode for a brief time before cytosolic Ca^2+^ levels increase. Local Na^+^ accumulation due to opening of voltage-gated Na^+^ channels could potentially contribute to CICR by increasing Ca^2+^ entry through NCX (Weber et al., 2003), but the very brief nature of these currents also questions their physiological relevance. Similarly, altered NKAα2 activity may create short-lived Na^+^ gradients that exert short-lived effects on NCX activity.

Several studies, however, suggest that the Na^+^ gradients are generated and maintained throughout several beats (Wendt-Gallitelli et al., 1993; Silverman, 2003). For example, we recently showed that several minutes of repetitive Na^+^ current activation increased the [Na^+^] sensed by the NKA, whereas 10 s of repetitive Na^+^ current activation had no effect on the [Na^+^] sensed by the NKA (Skogestad et al., 2019a), in line with previous findings (Silverman, 2003). Further, the subsarcolemmal Na^+^ gradient dissipated very slowly (Skogestad et al., 2019a), suggesting that a Na^+^ gradient between the subsarcolemmal space and bulk cytosol might be continuously present in the beating heart.

Collectively, these data suggest that NKAα2 can generate local Na^+^ gradients that are further maintained by an unknown mechanism. We speculate that NKAα2 exerts short-term and long-term control of local [Na^+^] and, hence, NCX activity, allowing the functional NKAα2/NCX complex to regulate Ca^2+^ entry, with proposed effects on CICR, and Ca^2+^ extrusion. The underlying mechanisms are yet to be demonstrated, but we consider the undisputed role of NKAα2 in regulating cardiac NCX activity as a clear indication of Na^+^ gradients in cardiomyocytes.

### Role of NKAα2 in Cardiac Disease

Ca^2+^ plays an essential and complex role in the development of cardiac disease. Reduced cytosolic [Ca^2+^] could contribute to the contractile deficit in heart failure (Eisner, 2014), while overload of Ca^2+^ leads to activation of detrimental Ca^2+^-dependent signaling pathways and promotes ventricular arrhythmias (Marks, 2003; Wehrens et al., 2005). As a regulator of intracellular [Ca^2+^], NKAα2 could play a role in the development of cardiac disease. Several studies have investigated the role of NKAα2 in cardiac hypertrophy and ventricular arrhythmias, and these results are briefly reviewed here.

#### Cardiac Hypertrophy

Compared to WT and NKAα1 overexpression, overexpression of NKAα2 attenuated cardiac hypertrophy 2, 10, and 16 weeks after pressure overload in mice (Correll et al., 2014). There were no differences in Ca^2+^-dependent pro-hypertrophic mechanisms, such as NFAT and CaMKII, but the mice with NKAα2 overexpression had faster NCX-dependent Ca^2+^ extrusion. The authors concluded that the anti-hypertrophic effect of NKAα2 overexpression likely was due to lowering of [Ca^2+^] and [Na^+^] in strategic compartments (Correll et al., 2014). On the other hand, Rindler *et al* found that cardiac-specific NKAα2 inactivation delayed the onset of cardiac hypertrophy following pressure overload but that outcomes were similar to control animals at later stages (Rindler et al., 2013).

These contradictory findings can be reconciled by considering the following complicating factors: 1) Genetic models are impure systems because genetic modification of one protein leads to several secondary changes with unpredictable effects. Mice with overexpression of either NKAα2 or NKAα1 have reduced levels of the other isoforms, and direct functional interpretation is thus difficult. In addition, the expression of PLM and the Ser-63 and Ser-68 phosphorylation were reduced in the NKAα2 overexpression mice (Correll et al., 2014). 2) It is possible that endogenous glycosides at least partly mediate the effect of NKAα1 and NKAα2 on cardiac hypertrophy (Blaustein et al., 2016; Blaustein, 2017). Mice with ouabain-sensitive NKAα1 (SWAP mice) had increased cardiac hypertrophy following pressure overload, a response that was abolished following sequestration of endogenous cardiac glycosides (Wansapura et al., 2011). Predicting the hypertrophic effect of altering the NKAα isoforms is not straightforward when considering the different affinity of cardiac glycosides towards NKAα1 and NKAα2 and the altered expression of NKAα isoforms in the genetically modified mice [(Blaustein, 2017). 3] Overexpression and reduction of NKAα2 are expected to have opposite effects on intracellular [Ca^2+^], with different short- and long-term effects on cardiac contractility and hypertrophy. Although there were no baseline differences, the heterozygous NKAα2 mice showed increased contractility in the first weeks following pressure overload (Rindler et al., 2013). While increasing Ca^2+^-dependent cardiac contractility could be temporarily beneficial, the consequences are potentially more dire over a longer time course (Lou et al., 2012). In contrast, the NKAα2 overexpression mice had lower Ca^2+^ transient amplitude and increased NCX-dependent Ca^2+^ extrusion compared to WT (Correll et al., 2014), which could exert beneficial effects by strategically lowering Ca^2+^ in specific domains involved in cardiac hypertrophy development.

#### Ventricular Arrhythmias

Reduced NKA activity increases intracellular [Na^+^], reduces NCX-mediated Ca^2+^ extrusion, increases intracellular [Ca^2+^], and increases the risk of triggered ventricular arrhythmias in hypokalemia (Eisner and Lederer, 1979; Aronsen et al., 2015; Pezhouman et al., 2015; Skogestad and Aronsen, 2018), digitalis toxicity (Gonano et al., 2011), and the Ankyrin B syndrome (Mohler et al., 2003; Mohler et al., 2005; Camors et al., 2012; Popescu et al., 2016). The increased intracellular [Ca^2+^] following reduced NKA activity increases the frequency of arrhythmogenic Ca^2+^ waves (Camors et al., 2012; Aronsen et al., 2015) but also activates CaMKII (Gonano et al., 2011; Pezhouman et al., 2015; Popescu et al., 2016), which further promotes arrhythmias by activating Na^+^ and Ca^2+^ currents (Hund and Mohler, 2015; Pezhouman et al., 2015). The specific role of NKAα2 in arrhythmias has been examined by two publications from our group. We found that hypokalemia increased Ca^2+^ transient amplitude and increased the frequency of Ca^2+^ waves, which was abolished following NKAα2 inhibition (Aronsen et al., 2015). We also studied the effect on intracellular [Ca^2+^] and cellular arrhythmias following disruption of NKA from Ankyrin B, a proposed mechanism for ventricular arrhythmias in the Ankyrin B syndrome (Mohler et al., 2003; Mohler et al., 2004; Mohler et al., 2005). NKA/Ankyrin B disruption increased NCX-sensed Na^+^, reduced Ca^2+^ extrusion through NCX, and increased the frequency of Ca^2+^ sparks and Ca^2+^ waves (Skogestad et al., 2019b), thus mimicking the phenotype from the Ankyrin B^+/-^ mice (Camors et al., 2012), and all effects were mediated by NKAα2 (Skogestad et al., 2019b). These data collectively suggest that NKAα2 might be an upstream node for arrhythmias, where altered NKAα2 activity could influence intracellular [Ca^2+^] and CaMKII activity downstream. Specific activation of NKAα2 might thus represent a future anti-arrhythmic target that warrants further investigation.
